# Methyl jasmonate modulates non-enzymatic antioxidant defenses in sugarcane under *Diatraea saccharalis* (Fabricius, 1794) infestation

**DOI:** 10.1186/s12870-025-07912-w

**Published:** 2025-12-19

**Authors:** Júlia Karoline Rodrigues das Mercês, Eduarda Gonçalves Reis, Milton Garcia Costa, Gustavo César Costa Gomes, João Rafael Silva Soares, Odair Aparecido Fernandes, Priscila Lupino Gratão

**Affiliations:** 1 School of Agricultural and Veterinary Sciences, Department of Biology, São Paulo State University (Unesp), Jaboticabal, 14884900 Brazil; 2 School of Agricultural and Veterinary Sciences, Department of Crop Protection, São Paulo State University (Unesp), Jaboticabal, 14884900 Brazil

**Keywords:** Sugarcane borer, Phytohormones, Resistance induction, Biotic stress, Defense priming

## Abstract

Sugarcane is exposed to several biotic stresses that limit productivity, with herbivory by the sugarcane borer *Diatraea saccharalis* (Fabricius, 1794) (Lepidoptera: Crambidae) being one of the most damaging. Larvae bore into stalks, disrupt sap flow, facilitate fungal and bacterial infections, and ultimately reduce plant performance and juice technological quality, while triggering an oxidative burst. In this context, defense priming offers a sustainable, cost-effective complement—and in some scenarios, an alternative—to insecticide-based control, by enhancing plant readiness rather than suppressing pests directly. The exogenous application of methyl jasmonate (MeJA) has emerged as a promising strategy to induce plants’ natural defenses by activating mechanisms similar to those triggered by pest attack. This study aimed to evaluate how exogenous MeJA modulates non-enzymatic antioxidant defenses in sugarcane under herbivory by *D. saccharalis* and under non-stress conditions. The experiment followed a randomized block design in a 2 × 4 factorial scheme with five replicates. The main factor was stress condition (with or without borer infestation), and the second factor was MeJA concentration (0, 0.25, 0.5, and 1 mmol L⁻^1^), applied seven days before infestation. Leaf samples were collected to assess lipid peroxidation [malondialdehyde (MDA)], non-enzymatic antioxidants (total phenolics, carotenoids, ascorbate and glutathione), total chlorophyll and anthocyanin index. Exogenous MeJA activated antioxidant defense pathways in both stressed and non-stressed plants in a dose- and context-dependent manner. Under borer infestation, the highest MeJA concentration (1 mmol L⁻^1^) enhanced defense responses, increasing phenolic compounds and carotenoids while significantly reducing MDA levels. In non-stressed plants, the lowest MeJA dose (0.25 mmol L⁻^1^) was more effective, lowering MDA and promoting the accumulation of phenolics, glutathione and anthocyanins. These results close an important gap on MeJA–sugarcane–borer interactions and suggest that different MeJA dose ranges could be strategically explored as complementary tools in integrated borer management, a hypothesis that now deserves validation under field conditions.

**Supplementary Information:**

The online version contains supplementary material available at 10.1186/s12870-025-07912-w.

## Introduction

Sugarcane (*Saccharum spp.*) is a major tropical crop, widely cultivated for its capacity to store sucrose and produce valuable by-products [1]. As sessile organisms, plants face continuous biotic challenges that alter cellular metabolism and impair growth [2]. Among these stresses, insects stand out for their abundance and diversity, constituting one of the main threats to plant survival [3].

From a mechanistic standpoint, herbivore attack triggers an oxidative burst—a rapid, transient increase in apoplastic reactive oxygen species (ROS) that functions as a defense signal but, when prolonged, can oxidize lipids, proteins, RNA, DNA, and small cellular molecules [4, 5]. Excess ROS is closely linked to lipid peroxidation and to the impairment of metabolic and photosynthetic processes [6]. In chewing herbivores, mechanical damage profoundly perturbs the extracellular space, releasing cell-wall fragments and intracellular components that intensify defense signaling [3].

Sugarcane borer, *Diatraea saccharalis* (Fabricius, 1794) (Lepidoptera: Crambidae), is the primary lepidopteran pest affecting sugarcane crops, significantly limiting plant growth and productivity [7]. Larvae bore into stalks, disrupt sap flow, facilitate fungal and bacterial infections, and ultimately reduce plant performance and technological quality of the juice [8, 9]. The sugarcane borer associated with phytopathogenic fungi is responsible for average sugar yield losses ranging from 0.52% and 1.10% per 1% of bored internodes [10]. In addition to physical damage and pathogen facilitation, *D. saccharalis* injury intensifies the oxidative burst, increasing the imbalance between ROS production and the plant's antioxidant capacity [6].

To constrain oxidative damage, plants mobilize enzymatic and non-enzymatic antioxidant systems. Ascorbate (AsA) and glutathione (GSH) are central to the AsA–GSH cycle, in which two molecules of AsA are used by ascorbate peroxidase (APX) to reduce hydrogen peroxide (H₂O₂) to water. AsA is then regenerated through a series of redox reactions involving GSH [11–13]. In addition, phenolic compounds and carotenoids also contribute to ROS quenching under stress [14, 15].

Although chemical control remains central in suppressing arthropod pest populations, the indiscriminate use of chemical pesticides can lead to well-known problems, such as pest outbreaks resulting from the selection of resistant populations [16]. In this context, defense priming offers a sustainable, cost-effective complement—and in some scenarios, an alternative—to insecticide-based control, by enhancing plant readiness rather than suppressing pests directly. This approach involves exposing plants to a trigger stimulus that transiently and partially activates the defense system, preparing them to respond more quickly and/or more intensely to future challenges [17, 18].

JA and its methyl ester, methyl jasmonate (MeJA), are the primary phytohormones involved in signal transduction in plants attacked by chewing insects [19], such as the sugarcane borer. Exogenous applications of MeJA act as defense elicitors in plants and have been associated with increases in metabolites with antioxidant functions, such as phenolics, carotenoids, and anthocyanins [20–22]. In sugarcane, foliar application of MeJA has been reported to trigger defensive responses against damage caused by *Chilo sacchariphagus indicus* (Lepidoptera: Crambidae) [23] as well as to increase plant resistance to *D. saccharalis* [16]. Nevertheless, how MeJA modulates non-enzymatic antioxidant defenses during *D. saccharalis* herbivory remains insufficiently resolved, particularly regarding the AsA–GSH cycle and dose–response relationships.

In this context, exogenous MeJA emerges as a promising strategy to induce plants’ natural defenses by activating mechanisms similar to those triggered under biotic stress, with emphasis on the AsA–GSH cycle, phenolics, and carotenoids. However, further investigation is needed into non-enzymatic antioxidant mechanisms—especially the AsA–GSH cycle, phenolics, and carotenoids—during herbivory by *D. saccharalis*, as well as dose–response relationships that would allow us to infer optimal MeJA concentrations for defense priming. We hypothesize that MeJA pretreatment increases non-enzymatic antioxidants and attenuates oxidative damage under borer attack relative to untreated plants. Thus, our objective was to evaluate how exogenous MeJA modulates non-enzymatic antioxidant mechanisms in sugarcane under *D. saccharalis* herbivory, with emphasis on the AsA–GSH cycle, phenolics and carotenoids.

## Materials and methods

### Experimental area characterization

The study was conducted in a greenhouse located in the experimental area of the Department of Crop Protection, Applied Ecology Sector, College of Agricultural and Veterinary Sciences, São Paulo State University (UNESP), Jaboticabal Campus, SP, Brazil (21°14′46" S, 48°18′39" W, and 600-m altitude). Maximum, average, and minimum relative humidity and temperature throughout the experimental period are presented in Supplementary Fig. 1.

### Treatments and experimental design

The experiment was arranged in a randomized block design with a 2 × 4 factorial scheme and five replications. Each replicate consisted of one plant per pot, totaling 40 experimental units, with each pot containing a plant with a single stalk. The first factor was the stress condition: with and without infestation by sugarcane borer (*D. saccharalis*). The second factor was the concentrations of methyl jasmonate (MeJA), applied at four levels: 0 mmol L⁻^1^ (without MeJA), 0.25 mmol L⁻^1^ (low), 0.5 mmol L⁻^1^ (medium), and 1 mmol L⁻^1^ (high). MeJA concentrations were selected based on previous sugarcane studies using MeJA as a biotic-stress modulator [16].

### Experiment installation and management

Soil samples from the A horizon were collected from an agricultural area with a history of peanut cultivation. These samples were then subjected to chemical analysis to evaluate soil fertility. Based on the results, pH correction and fertilization were performed according to crop requirements, following the fertilization and liming guidelines for crops in the state of São Paulo [24].

Sugarcane seedlings of the IACCTC07-8008 cultivar (considered susceptible) were produced using the Pre-Sprouted Seedling (MPB) technique [25], without the use of insecticides, and transplanted into 10 L pots containing soil. The plants were cultivated for 90 days after transplanting (DAT) before the foliar application of MeJA (this cultivar had not yet initiated tillering, so each plant consisted of a single stalk without the need for de-tillering). During the pre-application period, pot water status was maintained at 90% of field capacity (FC)—i.e., 90% of the substrate water held after free drainage—using a gravimetric approach: pots were weighed daily and irrigated to a target mass corresponding to 90% FC. FC for our substrate was determined prior to the trial by saturating the pots, allowing drainage to cease overnight, and recording pot mass; the 90% FC target was then calculated from this reference. Seven days after MeJA treatment, the plants were infested with *D. saccharalis* larvae.

The MeJA solution was prepared according to the treatment concentrations using a 1% methanol solution as a diluent. A stock solution of 1 mmol L⁻^1^ (high) MeJA was initially prepared, and working solutions of 0.25 (low) and 0.50 mmol L⁻^1^ (medium) were obtained by dilution; the 0.00 mmol L⁻^1^ control (without MeJA) consisted of the diluent only (1% methanol). Solutions were applied immediately after preparation using a manual sprayer, ensuring full coverage of the aerial parts of the plants until runoff. Each pot received 25.0 mL of solution, and only one foliar application was performed. Spraying was conducted in the morning between 7:00 and 8:00 a.m. During application, environmental conditions were monitored, with temperatures ranging from 20 to 25 °C and relative humidity above 70%, which are considered favorable for foliar absorption.

*D. saccharalis* larvae were obtained from the Entomology Laboratory, where they were reared on an artificial diet originating from the São Martinho farm in Pradópolis, São Paulo, Brazil (21°21′23′′S, 48°3′48′′W). We used third-instar larvae to ensure predictable boring within 24–48 h and lower-variance gallery-length measurements, following validated infestation protocols [26]. Three larvae were removed from the diet and were transferred and positioned with the help of a brush of thin hair close to the sheath of + 2 and + 3 leaves of each sugarcane plant subjected to the treatments, allowing for natural infestation. The method was adapted from Soares et al., [27]. Larval behavior was monitored hourly for six hours following placement, and plants were observed for three consecutive days to confirm successful infestation, indicated by stem boring.

### Biometric, physiological, and biochemical parameters

Plant height, number of leaves, and stem diameter were measured to standardize and verify the initial condition of the plants before treatment application. Seven days after infestation with *D. saccharalis* larvae, some plants began to exhibit symptoms of stem boring, such as leaf tip desiccation. At this stage, leaf sampling was initiated for biochemical analyses, which included the following parameters:

#### Photosynthetic pigments

Leaf disks measuring 26.4 mm^2^ were collected and processed under shaded conditions. The disks were placed in 80% acetone, and the resulting extracts were transferred to aluminum foil–wrapped tubes and stored under refrigeration for five days. Pigment concentrations were determined using a benchtop spectrophotometer at the following wavelengths: chlorophyll a at 663 nm, chlorophyll b at 647 nm, and carotenoids at 470 nm [28]. Total chlorophyll was calculated as the sum of chlorophyll a and b. The anthocyanin index was measured on the central adaxial surface of the second fully expanded leaf using a Multiple Pigment Meter (MPM-100, OPTI-Sciences, USA).

#### Lipid peroxidation

Fresh leaf tissue was macerated with 20% (w/v) polyvinylpyrrolidone (PVPP) and 0.1% trichloroacetic acid (TCA). The homogenate was centrifuged at 10,000 rpm for 15 min, and the supernatant was mixed with a solution containing 20% TCA and 5% thiobarbituric acid (TBA). The mixture was incubated in a water bath at 95 °C for 30 min. The reaction was stopped by placing the tubes in an ice bath for 10 min, followed by centrifugation at 10,000 × g for 15 min at 4 °C. The thiobarbituric acid reactive substances (TBARS) content was used to estimate lipid peroxidation. Malondialdehyde (MDA) concentration was calculated using an extinction coefficient of 1.55 × 10^5^ mol⁻^1^ cm⁻^1^, based on absorbance readings between 535 and 600 nm [29]. Results were expressed as μmol per mg of fresh tissue.

#### Non-enzymatic antioxidants

The total phenolic content was determined following a method adapted from Singleton; Rossi, [30]. Leaf disks (0.05 g fresh weight) were placed in 15 mL Falcon tubes wrapped in aluminum foil, and 2 mL of concentrated methanol was added. Tubes were sealed and incubated in a water bath at 25 °C for 3 h. After incubation, the disks were removed, and the extract was filtered. An additional 3 mL of methanol was then added. For the colorimetric assay, 1 mL of the filtered extract was transferred to a new 15 mL Falcon tube (also foil-wrapped), followed by the addition of 15 mL distilled water and 0.5 mL of 2 N Folin–Ciocalteu reagent. After a 3-min reaction period, 1.5 mL of sodium carbonate was added, and the mixture was allowed to stand for 2 h. Absorbance was then measured at 765 nm wavelength using a spectrophotometer.

Reduced ascorbate (AsA) content was determined as described by Kampfenkel; Van Montagu; Inzé, [31] with modifications. Leaf tissue (0.4 g) was homogenized in 2.0 mL of 6% (w/v) trichloroacetic acid (TCA) and centrifuged at 10,000 rpm for 10 min at 4 °C. Aliquots of 200 µL of the leaf extract were used. Total ascorbate (AsA + DHA) was quantified after the reduction of dehydroascorbate (DHA) using dithiothreitol (DTT). For this, 200 µL of extract was added to 200 µL of 10 mM DTT (in 0.2 M sodium phosphate buffer, pH 7.4) and 400 µL of the same buffer. The mixture was incubated at 42 °C for 15 min. After incubation, the following reagents were sequentially added: 100 µL of 0.5% (w/v) N-ethylmaleimide, 100 µL of 50% (w/v) TCA, 400 µL of 42% (v/v) H₃PO₄, 400 µL of 4% (w/v) 2,2′-dipyridyl (in 70% ethanol), and 400 µL of 3% (w/v) FeCl₃. The mixture was agitated and incubated at 42 °C for 40 min. The reaction was stopped by placing the tubes in an ice bath, and absorbance was read at 525 nm wavelength. Results were expressed as µmol g⁻^1^ fresh weight.

Glutathione (GSH) content was determined according to Griffith, [32], based on the sequential reduction of 5,5'-dithiobis (2-nitrobenzoic acid) (DTNB) by NADPH in the presence of glutathione reductase (GR). Approximately 0.2 g of fresh leaf tissue was homogenized in 100 mM phosphate buffer (pH 7.5) containing 0.5 mM EDTA. The reaction mixture (1 mL total volume) contained: 150 µL of 125 mM phosphate buffer with 6.3 mM EDTA (pH 6.5), 700 µL of 0.3 mM NADPH, 100 µL of 3 mM DTNB, and 50 µL of the sample extract. The reaction was initiated by adding 10 µL of GR, and absorbance was monitored at 412 nm.

#### Quantification of stalk injury

After the infestation period, stalks were split longitudinally with a sterilized blade to expose galleries. Each stem was photographed perpendicular to the surface against a dark background with a millimeter ruler in the same plane for scale. Images (JPEG, ≥ 300 dpi) were analyzed in ImageJ (v1.53): after scale calibration, the total stem area (Polygon) and gallery areas (Freehand) were delineated and measured. Percent injury was computed as %injury = 100 × (ΣGallery_area/Stem_area). When multiple images per stalk were available, values were averaged; the plot-level variable was the mean %injury of the sampled stem.

### Statistical analysis

Statistical analyses were performed using Python (version 3.9.7; Python Software Foundation). Data normality was assessed using the Shapiro–Wilk test, and homogeneity of variances was evaluated using Levene’s test to ensure consistent variance across groups. Subsequently, analysis of variance (ANOVA) was conducted to detect statistically significant differences (*p* < 0.05). When ANOVA indicated significance, Tukey’s post-hoc test was applied to identify specific group differences.

Hierarchical clustering was conducted using the *sns.clustermap* function from the Seaborn library, applying the average linkage method (method = 'average') and Euclidean distance metric (metric = 'euclidean') to assess similarity between samples. Data were standardized by column (*standard_scale* = 1), and the "*coolwarm*" colormap was used to represent data variation. Hierarchical clustering was performed exclusively on the biochemical variables (e.g., ascorbate/glutathione metrics, total phenolics, carotenoids, chl total, anthocyanins, MDA).

Principal component analysis (PCA) was also performed to explore the data structure and variability. Standard deviations of each variable were first calculated to assess dispersion. Variables were then standardized based on the covariance matrix. The PCA included all biochemical variables used in the hierarchical clustering (ascorbate/glutathione metrics, total phenolics, carotenoids, total chlorophyll, anthocyanins, MDA) and, additionally, the injury (%) variable. A biplot was generated to simultaneously visualize individuals and variables, with ellipses added to highlight groupings as a function of treatments.

## Results

### Growth analysis

**Fig. 1:**
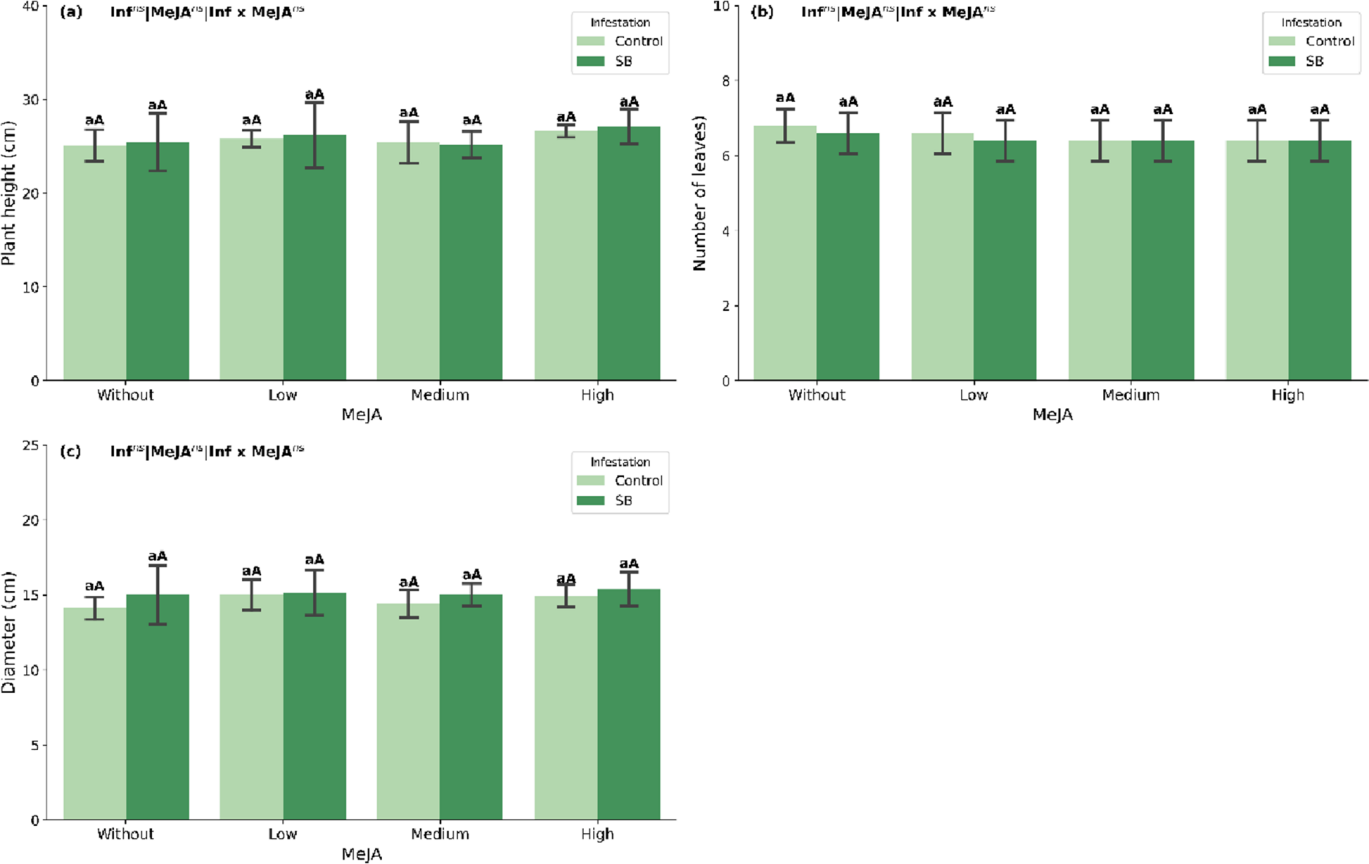
Plant height (**a**), number of leaves (**b**), and stalk diameter (**c**) of sugarcane plants (mean ± SEM) treated with methyl jasmonate (MeJA) under non-infested (Control) and sugarcane borer (SB)-infested conditions. Different lowercase letters indicate significant differences between infestation conditions; different uppercase letters indicate significant differences among MeJA concentrations (Tukey’s test, *p* < 0.05)

Before the experiment, plants were evaluated for height, leaf number, and stalk diameter. Consistent with ANOVA results (Supplementary Table S1), infestation, MeJA and their interaction did not significantly affect these parameters (*p* > 0.05), confirming the initial homogeneity of the experimental units (Fig. 1a–c).

### Lipid peroxidation and stalk injury

**Fig. 2:**
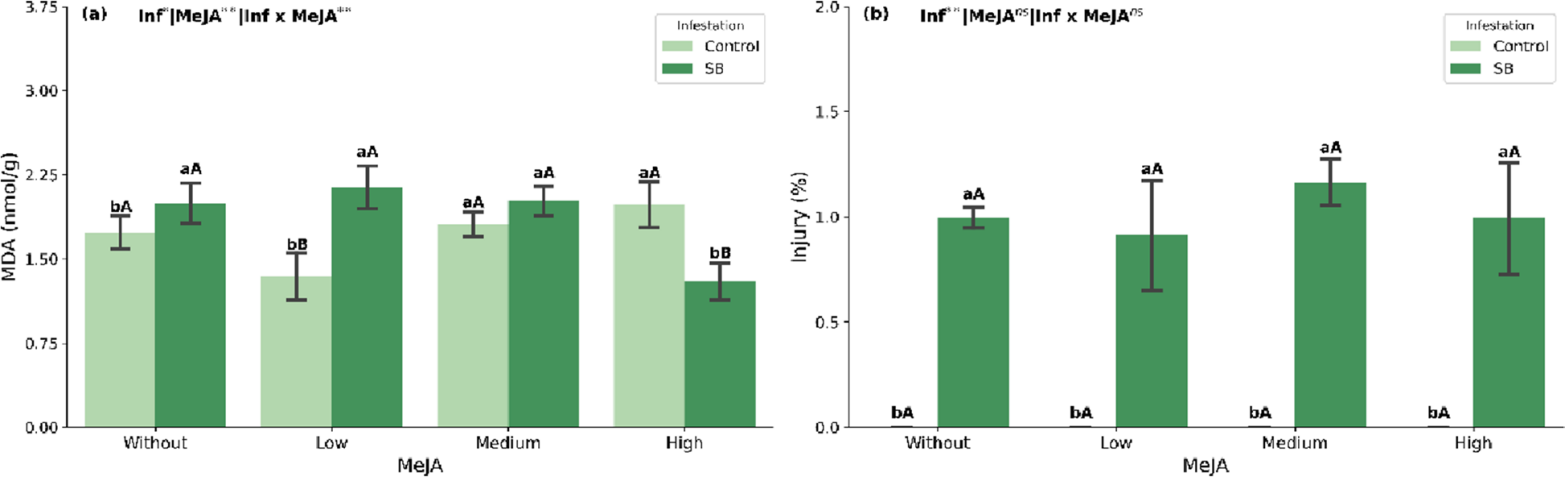
Malondialdehyde (MDA) content (**a**), Injury (%) (**b**) in sugarcane plants (mean ± SEM) treated with methyl jasmonate (MeJA) under non-infested (Control) and sugarcane borer (SB)-infested conditions. Different lowercase letters indicate significant differences between infestation conditions; different uppercase letters indicate significant differences among MeJA concentrations (Tukey’s test, *p* < 0.05)

According to the ANOVA (Supplementary Table S1), MDA content was significantly affected by infestation, MeJA dose and their interaction (*p* < 0.05), whereas stalk injury was influenced only by infestation. Sugarcane borer infestation increased malondialdehyde (MDA) content in plants not treated with MeJA and at the lowest MeJA concentration (Fig. 2), whereas the highest concentration (1 mmol L⁻^1^) significantly reduced MDA content (Fig. 2). Under infestation, the highest MeJA concentration reduced MDA content, while under non-stress conditions, a reduction in MDA was observed at the lowest MeJA concentration (Fig. 2a). Injury was absent in non-infested plants across all MeJA doses. Under infestation by *D. saccharalis*, injury remained similar across doses (≈1%) (Fig. 2b).

### Non-enzymatic antioxidant defense system

**Fig. 3:**
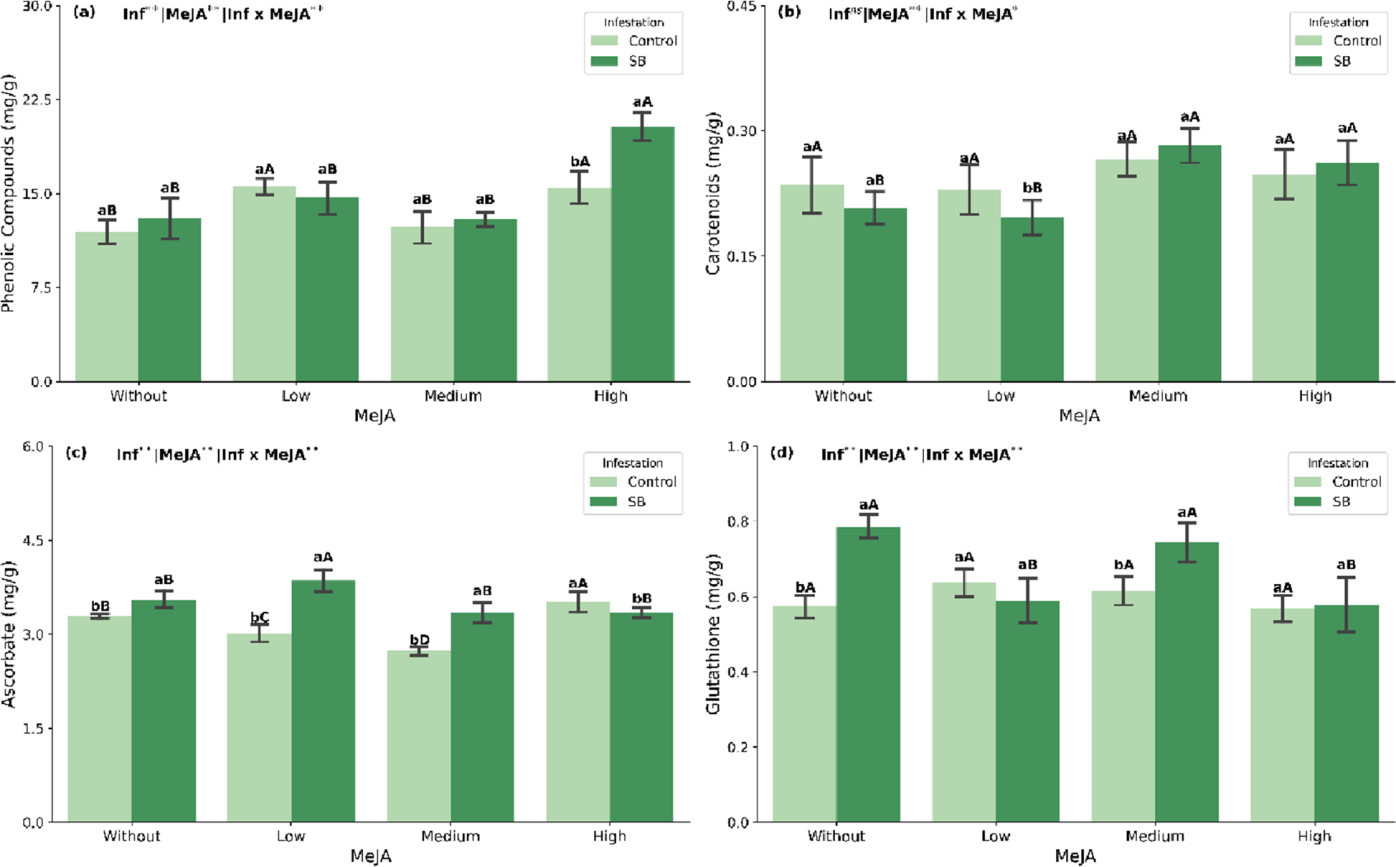
Phenolic compounds (**a**), carotenoids (**b**), ascorbate (**c**), and glutathione (**d**) in sugarcane plants (mean ± SEM) treated with methyl jasmonate (MeJA) under non-infested (Control) and sugarcane borer (SB)-infested conditions. Different lowercase letters indicate significant differences between infestation conditions; different uppercase letters indicate significant differences among MeJA concentrations (Tukey’s test, *p* < 0.05)

The ANOVA revealed significant main and/or interaction effects of infestation and MeJA dose on phenolic compounds, carotenoids, ascorbate, glutathione, total chlorophyll and anthocyanin index (Supplementary Table S1). Phenolic compound levels increased under sugarcane borer infestation only at the highest MeJA concentration (Fig. 3a). In non-stressed plants, phenolic content increased at both low and high MeJA concentrations (Fig. 3a). Carotenoid content in infested plants increased at medium and high MeJA concentrations, while no significant changes were observed across MeJA concentrations under non-stress conditions (Fig. 3b).

Ascorbate (ASA) content increased in response to borer infestation in plants not treated with MeJA and those treated with low and medium concentrations. In contrast, high MeJA concentration led to a reduction in ASA levels (Fig. 3c). When MeJA was applied under infestation, ASA levels increased at the low concentration, whereas in non-stressed plants, the highest ASA content was observed at the high MeJA concentration (Fig. 3c). For glutathione (GSH), content increased under infestation in both untreated plants and those receiving the medium MeJA concentration (Fig. 3d). Under infestation, the highest GSH level was recorded in plants treated with the medium MeJA dose. In non-stressed conditions, GSH content increased at low, medium, and high MeJA concentrations (Fig. 3d).

**Fig. 4:**
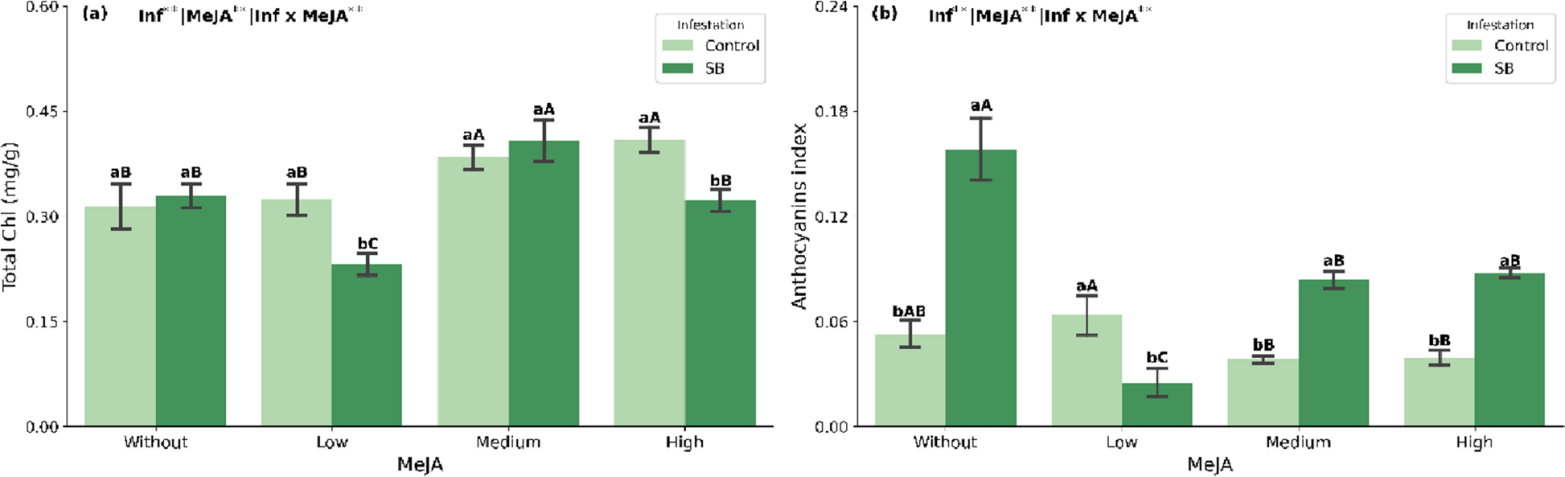
Total chlorophyll (Chl) (**a**) and anthocyanins (**b**) in sugarcane plants (mean ± SEM) treated with methyl jasmonate (MeJA) under non-infested (Control) and sugarcane borer (SB)-infested conditions. Different lowercase letters indicate significant differences between infestation conditions; different uppercase letters indicate significant differences among MeJA concentrations (Tukey’s test, *p* < 0.05)

Infestation by *D. saccharalis* reduced total chlorophyll content at both low and high MeJA concentrations (Fig. 4a). Under stress conditions, foliar application of MeJA increased total chlorophyll only at the medium concentration. In non-stressed plants, chlorophyll content increased at both medium and high MeJA concentrations (Fig. 4a).

Anthocyanin levels increased under borer infestation in plants that received no MeJA, as well as those treated with medium and high concentrations (Fig. 4b). However, under infestation, foliar application of MeJA resulted in an anthocyanin increase only in the absence of MeJA. In contrast, under non-stress conditions, anthocyanin content decreased at medium and high MeJA concentrations (Fig. 4b).

### Hierarchical clustering of response variables

**Fig. 5:**
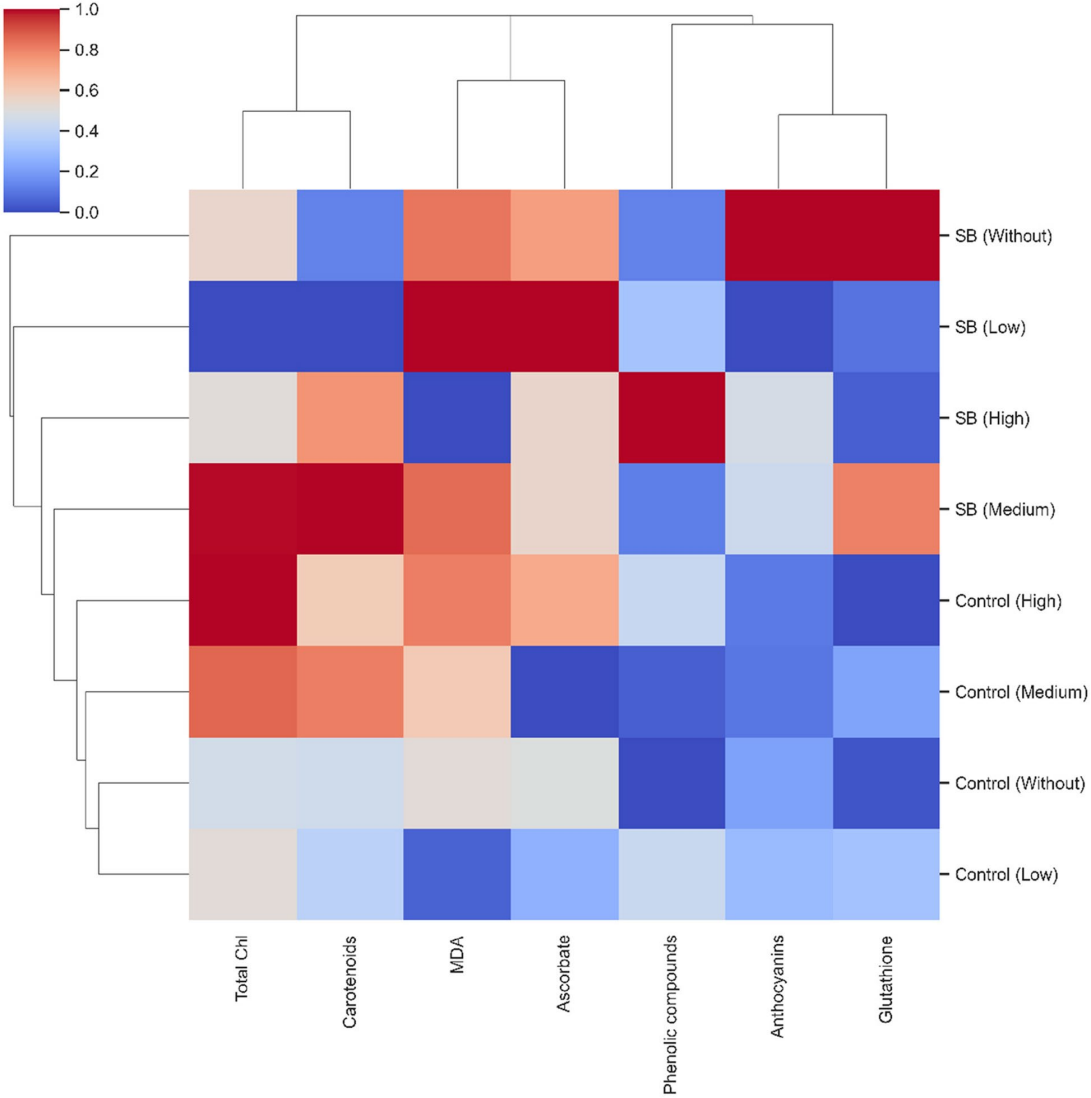
Clustering map of response variables: malondialdehyde (MDA), phenolic compounds, carotenoids, ascorbate, glutathione, Total chlorophyll (Chl), and anthocyanin index in sugarcane plants treated with methyl jasmonate (MeJA) under non-infested (Control) and sugarcane borer (SB)-infested conditions

Hierarchical clustering analysis revealed the formation of two main groups (Fig. 5). The first group consisted solely of plants infested by *D. saccharalis* without MeJA application, while the second encompassed all other treatment conditions. This second group was further divided into two subgroups: one comprising *D. saccharalis*-infested plants treated with the low MeJA concentration, and the other including plants treated with medium and high MeJA concentrations under infestation, along with all non-infested (control) plants (Fig. 5).

The analysis indicated a closer similarity between treatments showing elevated MDA levels, particularly *D. saccharalis*-infested plants without MeJA or with low MeJA concentration. High levels of anthocyanins and glutathione were exclusively associated with infestation in the absence of MeJA. The greatest increase in ascorbate was observed under *D. saccharalis* infestation combined with the highest MeJA concentration. Additionally, increased phenolic compound levels were linked to infestation with high MeJA concentration, whereas higher carotenoid levels were associated with infestation under medium MeJA concentration (Fig. 5).

### Principal component analysis (PCA)

**Fig. 6:**
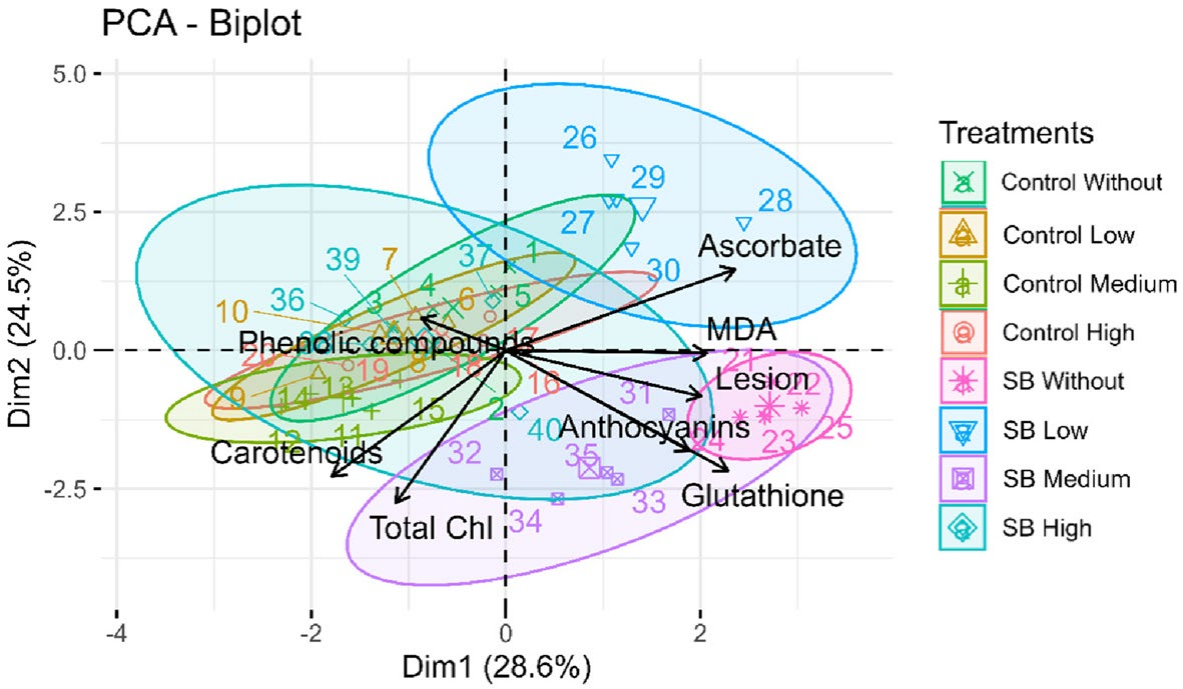
Principal component analysis of response variables: malondialdehyde (MDA), phenolic compounds, carotenoids, ascorbate, glutathione, Total chlorophyll (Chl), and anthocyanin index in sugarcane plants treated with methyl jasmonate (MeJA) under non-infested (Control) and sugarcane borer (SB)-infested conditions

Principal component analysis (PCA) revealed that the first two components explained 28.6% (Dim1) and 24.5% (Dim2) of the total variance, accounting for 53.1% overall (Fig. 6). The PCA showed that *D. saccharalis* infestation without MeJA application, as well as infestation with medium MeJA concentration, were associated with increased damage (Fig. 2b), and elevated levels of MDA, anthocyanins, and glutathione, along with reductions in chlorophyll and carotenoids. In contrast, *D. saccharalis* infestation combined with low MeJA concentration was associated with increased ascorbate levels (Fig. 6). Additionally, *D. saccharalis* infestation combined with the high MeJA concentration was linked to elevated contents of phenolic compounds (Fig. 6).

## Discussion

### Impact of borer infestation on lipid peroxidation and plant defense activation

Infestation by *D. saccharalis* in the absence of exogenous MeJA caused a clear redox imbalance in sugarcane. Infested plants without MeJA showed higher MDA levels than non-infested plants, indicating intensified membrane lipid peroxidation (Fig. 2a), even though the percentage of visible stalk injury remained low (≈1%; Fig. 2b). This pattern suggests that a relatively short feeding period by third-instar larvae was sufficient to trigger an oxidative burst before extensive tissue damage became apparent, which is consistent with reports that herbivory promotes rapid production of reactive oxygen species (ROS) and disrupts the balance between their generation and scavenging [33]. Excess ROS is closely linked to lipid peroxidation and to the impairment of metabolic and photosynthetic processes [6]. These results corroborate previous studies reporting increased lipid peroxidation when plants are exposed to adverse stress conditions [34–36].

To cope with stress, plants activate defense mechanisms that preserve cellular function, involving both enzymatic and non-enzymatic antioxidant systems [12, 13]. In infested plants without MeJA, higher AsA and GSH levels were observed compared with the non-infested control (Fig. 3c, d), indicating activation of the AsA–GSH cycle as a first line of defense against excess ROS. Ascorbate (AsA) and glutathione (GSH) are essential components of antioxidant protection and plant functioning, playing distinct yet complementary roles [11, 12].

In parallel, there was a marked increase in anthocyanin content in this treatment (Fig. 4b), reinforcing the role of this group of flavonoids as additional components of the antioxidant barrier under infestation. Anthocyanins are an important class of flavonoid pigments, and their biosynthesis can be induced in response to biotic stresses [37]. It was recently demonstrated in citrus that anthocyanins accumulate in fruits in response to fungal infection, preventing pathogen infestation [38]. Enhanced anthocyanin biosynthesis in tomato has been associated with increased resistance to *Botrytis cinerea* [39], reinforcing the role of these compounds in mitigating biotic stresses.

However, even with the simultaneous activation of the AsA–GSH cycle and anthocyanin biosynthesis, MDA levels remained high in infested plants without MeJA, indicating that this natural response was not sufficient to fully neutralize the oxidative stress imposed by *D. saccharalis*. Principal component analysis and hierarchical clustering support this interpretation, isolating the “SB without MeJA” treatment as the one most strongly associated with high MDA, GSH and anthocyanin levels and with lower chlorophyll and carotenoid contents (Figs. 5 and 6). This scenario represents the condition of greatest oxidative vulnerability in the system, upon which the modulatory effect of MeJA is superimposed in the following sections.

### Impact of MeJA application on lipid peroxidation and plant defense activation under borer infestation

When *D. saccharalis* infestation was combined with MeJA application, the antioxidant response became strongly dependent on the regulator dose. Under borer attack, the highest MeJA concentration tested (1 mmol L⁻^1^) significantly reduced malondialdehyde (MDA) content to values close to those observed in non-infested plants, whereas the lowest dose was not able to prevent the increase in MDA (Fig. 2a). This indicates that, under herbivory-induced oxidative stress, a stronger jasmonate signal is required to effectively limit lipid peroxidation.

The decrease in MDA in the presence of high MeJA was accompanied by a reconfiguration of antioxidant metabolites. In infested plants, the highest MeJA concentration promoted a pronounced accumulation of phenolic compounds (Fig. 3a) and an increase in carotenoids, especially at the high dose, with a 26% increase compared with the control (Fig. 3b). These two groups of compounds were the main markers of the “SB + high MeJA” treatment in the cluster map and PCA (Figs. 5 and 6), suggesting that, under infestation, MeJA redirects metabolism toward the phenylpropanoid pathway and carotenoid biosynthesis as central defense modules. Phenolics and carotenoids are well recognized as efficient ROS detoxifiers and protectors of photosynthetic structures, which partly explains the lower membrane peroxidation observed in this treatment.

Most phenolic compounds are synthesized via the shikimate pathway, where phenylalanine ammonia-lyase (PAL) catalyzes the conversion of phenylalanine to cinnamic acid, a precursor for a wide range of secondary metabolites, including phenolics [40]. MeJA is involved in plant signal transduction and has been shown to induce the expression of genes such as PAL, thereby stimulating phenolic biosynthesis through the phenylpropanoid pathway [41, 42]. Several studies have shown that MeJA application stimulates phenolic biosynthesis in plants under stress conditions [43, 44], which is in line with the increase in phenolics observed here in infested plants treated with the highest MeJA dose.

Carotenoids, in turn, are isoprenoids synthesized via the mevalonic acid pathway and play an essential role in photoprotection and dissipation of excess energy, thereby helping to attenuate oxidative damage [14, 15]. There is evidence that MeJA stimulates carotenoid biosynthesis by positively regulating key enzymes such as phytoene synthase (PSY) [34, 45–47]. An increase in carotenoid content in MeJA-treated plants has been widely documented in different species under stress. Similar trends were reported in maize under salinity stress [48] and in citrus, where JA promoted increases in carotenoids such as β-carotene, β-cryptoxanthin and violaxanthin [22]. Tomatoes treated with MeJA also exhibited higher production of lycopene, β-carotene and lutein [49].

AsA and GSH also responded to MeJA under infestation, but with more subtle dose-dependent patterns: the lowest dose was associated with higher AsA levels, whereas the medium dose promoted the highest GSH contents (Fig. 3c, d). However, these isolated increases were not sufficient to reduce MDA to the same extent observed with high MeJA, reinforcing the idea that, under borer attack, strengthening the phenylpropanoid and carotenoid pathways is more decisive for oxidative protection than a moderate enhancement of the AsA–GSH cycle.

Another relevant aspect is the shift in antioxidant strategy regarding anthocyanins. In the absence of MeJA, infested plants showed the highest levels of this pigment (Fig. 4b), indicating a strong reliance on this defense pathway. As the MeJA dose increased, the relative contribution of anthocyanins decreased, while phenolics and carotenoids became predominant in the antioxidant profile. Thus, high-dose MeJA not only intensifies the antioxidant response but also redistributes it among different classes of compounds, which appears to be more effective in restoring redox balance under *D. saccharalis* attack.

From an applied perspective, these results suggest that higher MeJA doses can enhance the ability of sugarcane to withstand oxidative bursts associated with borer damage and possible secondary infections. However, the experiment was conducted in pots, under controlled conditions and for a relatively short period, so potential impacts on growth, yield and sucrose accumulation under field conditions still need to be evaluated.

### Impact of MeJA application on lipid peroxidation and plant defense activation under non-stress conditions

Under non-infested conditions, MeJA application also modulated sugarcane redox status, but the response pattern differed from that observed under borer attack. The lowest MeJA dose reduced basal MDA levels compared with the MeJA-free control (Fig. 2a) and increased phenolic compounds, GSH and anthocyanins (Figs. 3a, d; 4b). This behavior is consistent with a “priming” effect, in which a low jasmonate dose raises antioxidant pools and places the plant in a state of greater readiness to respond to future stresses, while keeping oxidative damage at reduced levels [50].

Medium and high MeJA doses in non-infested plants also increased some antioxidant metabolites—particularly GSH at the medium dose and phenolics and AsA at the high dose (Fig. 3c, d)—and supported the maintenance of photosynthetic pigments, with increases in total chlorophyll relative to the control (Fig. 4a). However, these higher doses did not produce further reductions in MDA compared with the low dose (Fig. 2a), suggesting that, under optimal growth conditions, a jasmonate signal above a certain threshold may impose metabolic costs without proportional additional benefits in oxidative protection. This is consistent with the notion that ROS also act as important signaling molecules and that a fine balance between their production and scavenging is crucial for normal plant metabolismo [51, 52].

Lipid peroxidation is known to be a natural outcome of plant metabolism, driven by the continuous production of ROS. While commonly associated with stress, lipid peroxidation also occurs under normal physiological conditions in a controlled manner [53]. In this context, MeJA application—even under non-stress conditions—can help reduce MDA levels.

Cluster analysis showed that non-infested treatments, regardless of MeJA dose, grouped together with infested plants treated with medium and high MeJA (Fig. 5), characterizing an intermediate physiological state with moderate MDA and high antioxidant levels. This pattern suggests that MeJA application, even in the absence of herbivory, shifts plants into a “pre-activated” state similar to acclimation to moderate stress. From an agronomic standpoint, this highlights the potential of MeJA as a tool to prepare sugarcane for early *D. saccharalis* attacks, in which low doses could be explored as prophylactic sprays to enhance antioxidant capacity before pest arrival.

Overall, our findings suggest that exogenous MeJA application at low concentrations is effective in enhancing antioxidant compound synthesis and directly neutralizing ROS produced during regular metabolic activity. Moreover, elevated baseline levels of antioxidant compounds may improve the plant’s readiness to respond to stress events, increasing resilience under variable or adverse environmental conditions.

Despite these promising patterns, some limitations of this study should be acknowledged. The experiment was conducted in pots under controlled greenhouse conditions, involved a single cultivar and a relatively short infestation period, which may constrain extrapolation to commercial fields. Future work should validate these dose–response relationships under field conditions, across cultivars and developmental stages, and assess their consequences for yield, sucrose accumulation and compatibility with existing borer-control tactics.

## Conclusions

Exogenous methyl jasmonate (MeJA) modulated non-enzymatic antioxidant defences in sugarcane in a clearly dose- and context-dependent manner. Under *Diatraea saccharalis* infestation, protection against oxidative stress was associated with the highest MeJA dose (1 mmol L⁻^1^), which shifted metabolism towards phenolic compounds and carotenoids, whereas under non-stress conditions a low dose (0.25 mmol L⁻^1^) was sufficient to lower basal lipid peroxidation while enhancing antioxidant pools, consistent with defence priming. These results close an important gap on MeJA–sugarcane–borer interactions and suggest that different MeJA dose ranges could be strategically explored as complementary tools in integrated borer management, a hypothesis that now deserves validation under field conditions.

## Supplementary Information

Supplementary Material 1.

## Data Availability

The datasets used and/or analyzed during the current study are available to the corresponding author upon reasonable request.
