# Supplementary material for: Methyl jasmonate modulates non-enzymatic antioxidant defenses in sugarcane under Diatraea saccharalis (Fabricius, 1794) infestation
Source: BMC Plant Biol. 2025 Dec 19;26:126. doi: 10.1186/s12870-025-07912-w (PMC12831377; doi:10.1186/s12870-025-07912-w)
Supplement: Supplementary file 1 — Supplementary Material 1. [file 12870_2025_7912_MOESM1_ESM.docx]

**Supplementary Material**

**Fig. 1.** Maximum and minimum values of temperature and relative humidity throughout the greenhouse experiment.

**
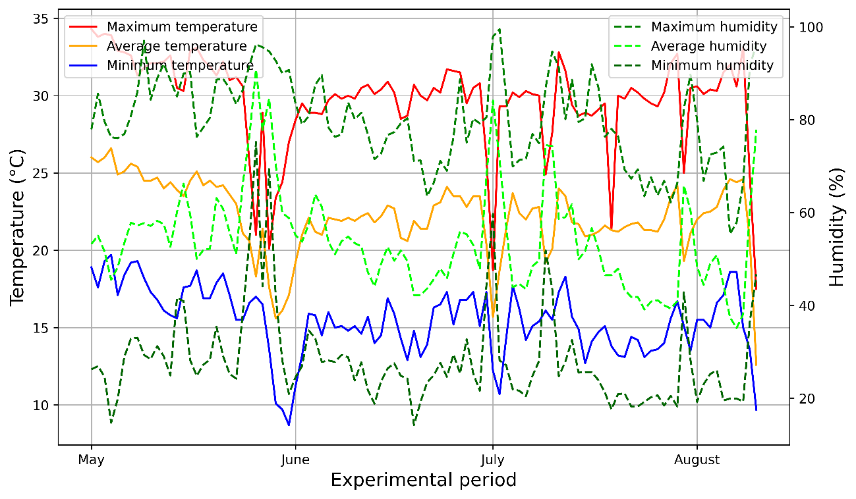
**

**Table 1.** Analysis of variance (F and P values) response variables: plant height, number of leaves, diameter, malondialdehyde (MDA), injury, phenolic compounds, carotenoids, ascorbate, glutathione, chlorophyll total and anthocyanin index in sugarcane plants treated with sugarcane borer (SB)-infested conditions and methyl jasmonate (MeJA).

| **Variables** | **CV%** | **-------------- F test --------------** | | | **-------------- P value--------------** | | |
| --- | --- | --- | --- | --- | --- | --- | --- |
|  |  | **Infestation** | **MeJA** | **Inf**  *****  **MeJa** | **Infestation** | **MeJA** | **Inf**  *****  **MeJa** |
| Plant height | 7.29 | 0.17 ^ns^ | 1.56 ^ns^ | 0.08 ^ns^ | 0.6857 | 0.2208 | 0.9728 |
| Number of leaves | 7.38 | 0.43 ^ns^ | 0.87 ^ns^ | 0.14 ^ns^ | 0.5154 | 0.4692 | 0.9322 |
| Diameter | 7.16 | 2.33 ^ns^ | 0.71 ^ns^ | 0.23 ^ns^ | 0.1381 | 0.5542 | 0.8749 |
| MDA | 9.68 | 7.04 **^*^** | 5.02 **^**^** | 31.30 **^**^** | 0.0130 | 0.0065 | 0.0001 |
| Injury | 26.19 | 583 **^**^** | 1.56 ^ns^ | 1.58 ^ns^ | 0.0001 | 0.2155 | 0.2155 |
| Phenolic Compounds | 8.33 | 13.51 **^**^** | 44.45 **^**^** | 10.21 **^**^** | 0.0010 | 0.0001 | 0.0001 |
| Carotenoids | 9.98 | 0.99 ^ns^ | 14.21 **^**^** | 3.03 **^*^** | 0.3276 | 0.0001 | 0.0460 |
| Ascorbate | 3.83 | 90.63 **^**^** | 23.62 **^**^** | 29.89 **^**^** | 0.0001 | 0.0001 | 0.0001 |
| Glutathione | 7.46 | 25.55 **^**^** | 12.21 **^**^** | 15.25 **^**^** | 0.0001 | 0.0001 | 0.0001 |
| Chl total | 5.85 | 30.44 **^**^** | 67.78 **^**^** | 24.92 **^**^** | 0.0001 | 0.0001 | 0.0001 |
| Anthocyanins index | 12.69 | 212 **^**^** | 89.37 **^**^** | 115.66 **^**^** | 0.0001 | 0.0001 | 0.0001 |
|  |  |  |  |  |  |  |  |

Inf = infestation (with or without sugarcane borer); MeJA = methyl jasmonate dose (0, 0.25, 0.5 and 1 mmol L⁻¹); Inf × MeJA = interaction between infestation and MeJA; CV = coefficient of variation. *, ** indicate significance at p ≤ 0.05 and 0.001, respectively; ns = not significant (p > 0.05).
